# 1822. Clinical Outcomes Associated with Oral Beta-Lactam Therapy versus Oral Fluoroquinolones/TMP-SMX for Uncomplicated Gram-Negative Bacteremia

**DOI:** 10.1093/ofid/ofac492.1452

**Published:** 2022-12-15

**Authors:** Satwinder S Kaur, Jessica T Babic, Steven Nguyen, Shivani Patel

**Affiliations:** Texas Health Harris Methodist Hospital Fort Worth, Dallas, Texas; Memorial Hermann-Texas Medical Center, Houston, Texas; Memorial Hermann, Houston, Texas; Memorial Hermann, Houston, Texas

## Abstract

**Background:**

Agents commonly used as oral step-down therapy in *Enterobacterales* bacteremia include fluoroquinolones and TMP-SMX. The use of these oral agents have been associated with adverse events. Oral β-lactams are not recommended to treat *Enterobacterales* bacteremia because of concerns for sub-therapeutic serum concentrations. Given the limited data on clinical effectiveness of oral β-lactams and increased limitations with fluoroquinolones and TMP-SMX, oral β-lactams may be an alternative option. The purpose of this study is to evaluate the clinical effectiveness in patients transitioned to oral β-lactams versus fluoroquinolones or TMP-SMX for the step-down treatment of uncomplicated *Enterobacterales* bacteremia.

**Methods:**

A multi-center, retrospective, propensity-score matched, observational cohort study was conducted from July 1, 2017 to December 31, 2020, at 11 Memorial Hermann Health System hospitals among 690 patients with uncomplicated *Enterobacterales* bacteremia. Patients were included if they received > 1 day of intravenous antibiotic therapy followed by definitive oral step-down therapy with a β-lactam, fluoroquinolone, or TMP-SMX. The primary outcome was treatment failure within 30 days of starting oral antibiotics, defined as at least one of the following: recurrent bacteremia, transition to IV antibiotics, new-onset sepsis, 30-day readmission, or 30-day all-cause mortality.

**Results:**

After the patients were propensity score matched 1:1, a total of 199 patients with uncomplicated *Enterobacterales* bacteremia were included in each group. Treatment failure occurred in 8 patients (4%) who received β-lactams and 10 patients (5%) who received fluoroquinolones or TMP-SMX (OR, 0.79 [95% CI, 0.306-2.04] *p*-value 0.629). Median length of hospital stay was 5 days for both groups (*p*-value 0.911), and median duration of oral antibiotics was 10 days in the fluoroquinolone or TMP-SMX group and 11 days in the β-lactam group (*p*-value 0.377).

**Conclusion:**

Based on the results of this study, no significant difference in treatment failure was seen in patients who were transitioned to β-lactams versus fluoroquinolones or TMP-SMX as oral step-down therapy for uncomplicated *Enterobacterales* bacteremia. Oral β-lactams appear to be a safe and effective option.

**Disclosures:**

**All Authors**: No reported disclosures.

